# Bardet-Biedl syndrome, renal transplant and percutaneous nephrolithotomy: a case report and review of the literature

**DOI:** 10.4076/1757-1626-2-6771

**Published:** 2009-07-07

**Authors:** Seshikanth Middela, Konstantinos Polizois, Alison J Bradley, Poduri N Rao

**Affiliations:** Department of Urology, University Hospital of South Manchester Foundation NHS trust, Wythenshawe HospitalSouthmoor Road, Manchester, M13 0DPUK

## Abstract

Bardet-Biedl syndrome is an autosomal recessive disorder with obesity, polydactly, retinitis pigmentosa, hypogenitalism, intellectual impairment and varying degree of renal abnormalities. Fewer than ten cases of paediatric renal transplantation for BBS have been reported in literature so far. This is the only case report of BBS transplant urolithiasis which was dealt with percutaneous nephrolithotomy and has been stone free for seven years. This is a complex case with a rare genetic disorder, renal transplant, renal stone, ileal conduit, long loop and inversely placed kidney. This case exemplifies the need for multidisciplinary management of complex cases and emphasises PCNL as the safe method.

## Introduction

Bardet-Biedl syndrome (BBS) is an autosomal recessive disease characterised by polydactyly, retinal dystrophy, marked central obesity, mental retardation with structural and functional renal abnormalities often leading to end stage renal disease (ESRD) in 5-30% of patients [1,2] often needing transplantation [2-5]. However, transplantation is complicated by stone disease in 0.2% to 1.7% of the adult patients and 0.6% to 2.5% of the paediatric patients as reported in various series [6,7]. We report the only case of child with BBS who underwent renal transplantation and subsequently developed a large pelvic stone in the transplant kidney (TK). The stone was successfully treated by percutaneous nephrolithotomy (PCNL) as it was the only safe mode of treatment in this case. The case report highlights the various difficulties in treating a stone in TK and emphasises PCNL as the safe modality of treatment in these complex cases.

## Case presentation

**Figure 1.**
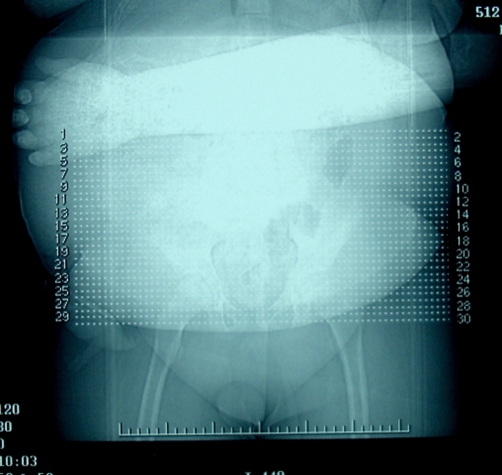
X ray of the hands.

A child, aged 14 years, weighing 115 kgs (Figure 1) was referred to our tertiary referral centre with an asymptomatic pelvic stone in his transplant kidney and a borderline renal function (creatinine of 115 µmol/l).

**Figure 2.**
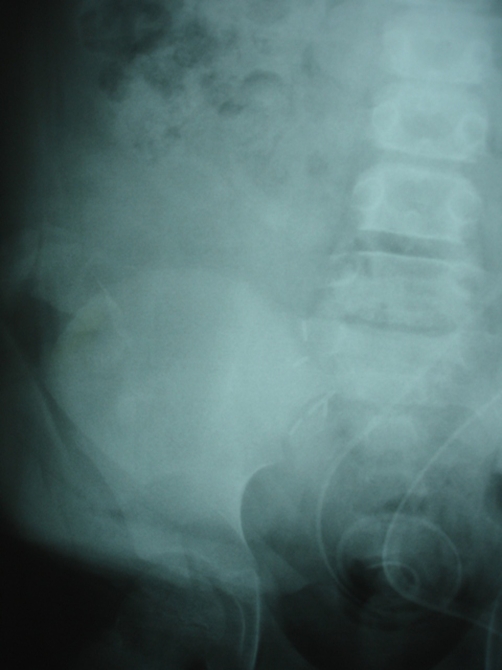
Immediate post operative X ray showing no stone.

His background problems included behavioural issues, neuropathic bladder with open bladder neck and marked obesity with sleep apnea. He needed socio-educational support and home nocturnal oxygen in the form of continuous positive airway pressure (CPAP). At the age of seven years he underwent Ileal diversion in view of his bladder dysfunction. He was finally diagnosed with Bardet-Beidl syndrome with all the characteristic features except syndactly (Figure 1). He gradually developed interstitial nephritis which progressed to ESRD. At the age of twelve, renal transplantation was offered and bilateral native nephrectomies were performed. Cadaveric renal transplantation was done in the right iliac fossa with ureteric implantation into the ileal conduit that was complicated with urinary leak. He had to undergo exploration and reimplantation the next day. The immediate postoperative imaging did not show any presence of stone (Figure 2).

**Figure 3.**
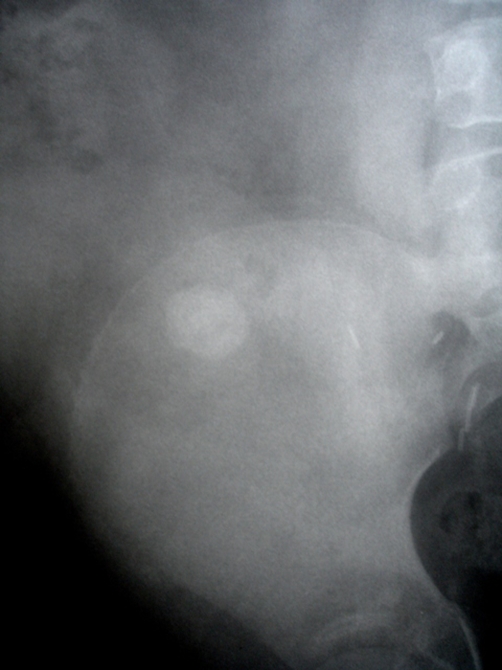
X ray two years later showing a 2 cm stone in the renal pelvis overlying pelvic bone.

**Figure 4.**
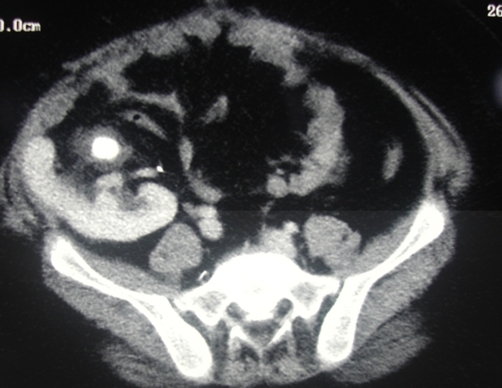
CT showing an inversely placed kidney with stone in the renal pelvis.

Postoperatively he was monitored by serial ultrasound scans and a year following the transplant a 2 cm stone (Figure 3) was identified in the renal pelvis though the patient himself was asymptomatic. Arrangements were made and put in place which specifically addresses his special needs. All the three modalities of treatment namely extracorporeal shockwave lithotripsy (SWL), flexible ureteroscopy (URS) and Percutaneous nephrolithotomy (PCNL) were considered. To determine delineate the anatomy and plan the best approach a CT scan of the pelvis and a Loopogram were organised. CT demonstrated a 2 cms stone and also showed that the kidney itself was placed in an inverted position (Figure 4).

**Figure 5.**
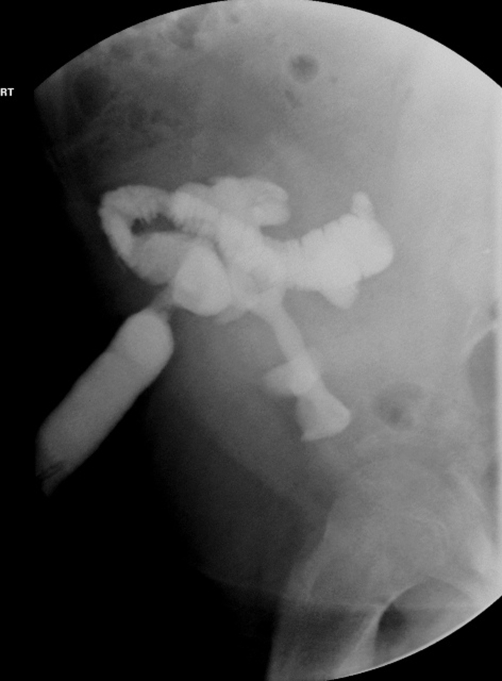
Loopogram showing a long tortuous loop overlying the kidney.

SWL was considered difficult due to his behavioural problems, marked obesity, sleep apnea requiring oxygen, large stone requiring more than one SWL sessions and the stone position. An alternative approach would have been a flexible URS with gravity aiding the fragments to fall into the conduit, however the loopgram showed a long tortuous loop (Figure 5) which effectively ruled it out. Loopogram did not delineate the transplant ureter as the loop itself was lying over the kidney. Finally it was decided that a percutaneous approach is the best option, given the circumstances.

The operation was performed on a Left Posterior Oblique Position. The renal access was gained under ultrasound guidance into the upper pole calyx (avoiding the overlying bowel loops) by an experienced Uroradiologist. A balloon dilator was used to dilate the tract and the stone was completely fragmented with an ultrasound lithotripter. At the end of the operation a nephrostomy tube was left in situ and the young patient was transferred to the paediatric high dependency unit for observation. Postoperative KUB showed no residual calcification. The recovery was uneventful, the nephrostomy was removed two days later and the patient was discharged the following day. He is stone free seven years after his PCNL based on his serial surveillance ultrasound scan with a normal creatinine (85 µmol/l).

## Discussion

BBS is an autosomal recessive disorder with obesity, polydactyl, retinitis, hypogenitalism, intellectual impairment and renal abnormalities. The prevalence in Europe and North America ranges from 1:14,000 to 1:16,000 live births while in the United Kingdom it is around 1: 125,000 live births [1,2]. They often progress to ESRD which may necessitate pre-emptive renal transplantation [2]. Fewer than ten cases of paediatric renal transplantation for BBS have been reported in literature so far. This is the only case report of BBS transplant urolithiasis which was dealt with PCNL and has been stone free for four years. This case exemplifies the need for multidisciplinary management of complex cases and emphasises PCNL as the safe method of treating these cases.

Unique anatomy, solitary precious kidney, absence of pain can delay the diagnosis of urolithiais and often make it difficult. It places them at higher risk of complications as compared to general population [8,9]. Our case was at more risk as compared to other paediatric TK because of a genetic disorder, long ileal loop and inversely placed kidney. Irrespective of the cause, the main aim of treating the stones in precious TK is preservation of the kidney and its function. Correction of metabolic abnormalities and treatment of recurrent urinary tract infections is needed in addition to definitive treatment.

Ben Challacombe et al [10] advocate adoption of the same aggressive protocol as that of treatment of stones in a solitary kidney to minimise the loss of function. SWL has been used to treat the stones however there many factors to be considered like patients compliance, availability of the service, operator skill etc. In addition SWL may fail to localise the stone due to overlying bowel or iliac bone [8-11]. SWL may require more than one sessions, has limited stone clearance and the risk of post-SWL steinstrasse which can be difficult to diagnose and manage [8,9]. They also will need some ancillary procedure like flexible URS or preoperative stenting [10]. In our case it was patient compliance (non-cooperation), respiratory impairment (need for oxygen) and his size (cannot lie prone) which precluded him from SWL treatment.

Flexible URS is a challenging option because of an ectopic anterior ureteric orifice, and ureteral fibrosis which makes ureter less pliable [8-11]. In the hands of a skilled surgeon and good flexible instruments the size of the stone seems to be the only limiting factor. However injury to the relatively devascularised ureter is a possibility and the healing may be delayed [11]. Anatomical variants like long ureter [11] and long ileal loop like ours may be encountered which make the standard ureteroscopes fall short of the target. Ureteric strictures may be encountered which increase the risk of ureteric injury and subsequent failure of procedure.

PCNL for TK seems to be the most reasonable option with many advantages over the other modalities. Achieving complete stone clearance is its single most advantage [9]. Most of the literature was limited to case reports until 2008, when there were series published by University of California and Mayo Clinic. Though small numbers (total 28 patients) they have demonstrated 100% stone clearance with minimal complications [8,12]. In fact some of the centres like Mayo clinic prefer PCNL because of its success rates [12]. Many recommend preoperative CT scan to delineate the complex anatomy around the TK and facilitate placement of the track around the bowel loops [8,9]. Our CT scan did show that the kidney was placed inversely. Access is usually placed by a radiologist under ultrasound guidance rather than fluoroscopy to avoid bowel injury [9]. TK is easy to access because it is pelvic and superficial [8,11]. However marked perirenal inflammation and fibrosis may limit the dilatation of the tracks and increase the risk of bleeding [8,9,11,12]. The inflammation will be more marked if there were urinary leak or re-implantations performed. It is in these cases alternative methods and techniques have been suggested such use of flexible scopes, various dilators, ice slush, open surgery etc [8,9,11].

Open surgery is recommended as the last option and a balance has to be weighed between the stone extraction and complications. Open surgery and reimplantation has been suggested where there is a coexisting stricture [11]. However it may be technically difficult due to intense perirenal scar tissue. Impaired wound healing and sepsis due to immunosuppression should be kept in mind for both PCNL and open surgery [8,9].

## Conclusion

Though the technique of PCNL in a transplant kidney is not different from that of a normal kidney, renal access is challenging and depending on the transplantation-related anatomical alterations, it may be the only option. In the hands of an expert it is a safe and effective modality in achieving stone free status. However these are complex cases requiring multidisciplinary approach, proper planning and tertiary experience in the surgical management of Urolithiasis.

## Abbreviations

BBS: Bardet-Biedl syndrome
CPAP: Continuous positive airway pressure
CT: Computed tomography
ESRD: end stage renal disease
PCNL: Percutaneous nephrolithotomy
SWL: Shockwave lithotripsy
TK: Transplant kidney
USR: Flexible ureteroscopy
